# Coagulopathy is associated with multiple organ damage and prognosis of COVID-19

**DOI:** 10.17179/excli2020-2853

**Published:** 2021-01-25

**Authors:** Rui Zhang, Yani Liu, Bei Zhang, Congqing Wu, Jinping Zhou, Yu Zhang, Wei Yang, Zhenyu Li, Shaojun Shi

**Affiliations:** 1 Department of Pharmacy, Union Hospital, Tongji Medical College, Huazhong University of Science and Technology, Wuhan 430022, PR China; 2 College of Public Health, Shaanxi University of Chinese Medicine, Xianyang 712046, PR China; 3 Saha Cardiovascular Research Center, College of Medicine, University of Kentucky, Lexington, KY40536, USA; 4 Department of Nutrition and Food Hygiene, Hubei Key Laboratory of Food Nutrition and Safety, School of Public Health, Tongji Medical College, Huazhong University of Science and Technology, 13 Hangkong Road, Wuhan 430030, PR China

**Keywords:** COVID-19, coagulopathy

## Abstract

Mortality rate is high with COVID-19. Multiple organ damage is a common and lethal complication of the severe COVID-19 patients. Of 198 recruited participants, 65 patients (32.8 %) had coagulopathy. In this retrospective study, we analyzed the association of coagulopathy with organ dysfunction in hospitalized COVID-19 patients. The incidence of coagulopathy was associated with increased odds of acute liver injury, renal dysfunction and acute respiratory distress syndrome (ARDS) by multivariable regression. Overall mortality was 65 % for the patients with coagulopathy and 3.76 % for the patients without coagulopathy. History of hypertension, leukocytosis and elevated CRP concentrations were associated with higher odds of coagulopathy. Patients with coagulopathy had similar levels of hepatic and renal functional enzymes prior to the onset of coagulopathy as the patients without coagulopathy, suggesting that coagulopathy is an association of the progression of multi-organ dysfunction in COVID-19. Plasma IL-6 was higher in patients with coagulopathy than controls, but it's not a risk factor for organ dysfunction by logistic regression. The present study shows that coagulopathy, overt DIC and non-overt DIC, associates with organ dysfunction and higher mortality rate in COVID-19. Thus, anticoagulant therapy may prevent organ dysfunction and increase survival rate in COVID-19.

## Introduction

COVID-19 is a world pandemic. As of April 22, 2020, it has caused 185,000 deaths with more than 2.6 million confirmed cases worldwide. Clinical symptoms of COVID19 ranged from common cold to fatal respiratory failure and death. Although most patients had mild symptoms and favorable prognosis, older patients, especially those with chronic comorbidities, suffered from severe outcomes such as ARDS and multi-organ dysfunction.

There is a growing evidence that coagulopathy and thrombosis are common complications in COVID-19, especially in non-survival COVID-19 patients (Tang et al., 2020[18]; Chen et al., 2020[3]; Zhou et al., 2020[28]; Guan et al., 2019[8]). Cao et al. first reported that prothrombin time and D-dimer levels are higher in severely ill patients (Huang et al., 2020[10]). Sun et al. reported that the D-dimer and prothrombin time are positively correlated with 28-day mortality in COVID-19 patients, while the platelet count is negatively correlated with mortality (Tang et al., 2020[17]). Disseminated intravascular coagulation (DIC) and multi-organ dysfunction (Chen et al., 2020[3]) are also confirmed in COVID-19 patients, and the reported probability of occurrence of DIC is 71.4 % for non-survivors (Tang et al., 2020[18][17]). In addition to DIC, another coagulation dysfunctional disease, venous thromboembolism (VTE), is also investigated in COVID-19 patients. The incidence of VTE in severe COVID-19 patients is 25 % and is related to poor prognosis (Cui et al., 2020[5]). Therefore, heparin (Tang et al., 2020[17]) as an anticoagulant treatment has been recommended in the early stage of the COVID-19 to prevent continuous increasing of inflammatory cytokines and D-dimer. 

DIC is manifested by the excessive thrombotic deposition in the microvasculature, which is thought to help entrap pathogens in blood flow. However, blood clots and fibrin deposition in blood vessels would also hamper oxygen delivery to the organs, and thus is an independent and powerful predictor of mortality in systemic infections. Multi-organ dysfunction is also a common and lethal complication in COVID-19 (Chen et al., 2020[3]). Many mechanisms may contribute to the development of multi-organ dysfunction in COVID-19, including inflammatory response (cytokine storm), shock, etc. However, the complete pathological process and contribution of DIC to COVID-19 progression have not been fully elucidated. In this study, we retrospectively analyzed 198 cases of COVID-19 patients admitted to Wuhan Union Hospital, with an aim to explore the relationship between organ dysfunction and coagulopathy. The dynamic changes of laboratory results including systemic inflammation in COVID-19 patients with and without DIC were also discussed. 

## Methods

### Study design and participants 

In the current retrospective cohort study, we included patients with confirmed COVID-19 who were hospitalized in the Department of Respiratory Medicine, Department of Infectious Diseases and ICU of Wuhan Union Hospital and had either discharged or had died from February 1, 2020 to March 4, 2020. Patients enrolled in this study met the following criteria: (1) diagnosed with COVID-19 according to the Chinese Clinical Guidance for COVID-19 Pneumonia Diagnosis and Treatment (China National Health Commission, 2020[4]); (2) aged between 18 to 90 years old; (3) had no history of liver and/or renal impairment; (4) had received both biochemical examinations (including liver and kidney functions) and coagulation tests (including prothrombin time, D-dimer antithrombin activity and fibrin degradation) during hospitalization. This study was performed in accordance with the Declaration of Helsinki (as revised in Brazil, 2013) (https://www.wma.net/policies-post/wma-declaration-of-helsinki-ethical-principles-for-medical-research-involving-human-subjects/) and approved by the independent ethics committee of Wuhan Union Hospital, Wuhan, China [approval no. (2020)-(0092)].

### Data collection 

A standardized data collection form and entry rules were used for extracting epidemiological, demographic, clinical, laboratory and outcome data from electronic medical records. All data were processed by two independent researchers (the collection and verification of information were never assigned to the same researcher), and errors and omissions were corrected and filled as necessary. A third researcher adjudicated any difference in interpretation between the two primary researchers. 

### Definitions

After excluding 133 patients without complete data of biochemical examination or coagulation tests, we included 198 moderately to severely ill patients in the current analysis. According to the ISTH) diagnostic criteria for DIC, patients were divided into 3 groups: overt-DIC patients (total points ≥ 5), non-overt DIC (total points between 3 to 4), and non-coagulopathy patients (total points ≤ 2). Coagulopathy patients included non-overt DIC patients and overt DIC patients. Acute liver injury was identified according to Schiff's diseases of the liver (Curry and Jeffers, 2017[6]) that the level of alanine aminotransferase exceeds twice the upper limits of normal (ALT > 80 U/L) (Curry and Jeffers, 2017[6]). Abnormal renal function was defined as serum levels of kidney creatinine were above upper limit of normal value (133 μmol/L).

### Laboratory procedures 

Methods for laboratory confirmation of SARS-CoV-2 infection have been described in the Guidance (http://www.gov.cn/zhengce/zhengceku/2020-02/05/content_5474791.htm), including respiratory specimens detection by next-generation sequencing/real-time RT-PCR methods, or imaging characteristics of pneumonia. Routine blood examinations, coagulation profile, serum biochemical tests (including renal and liver functional enzymes), cytokine storm examination were tested. Chest radiographs or CT scans were also performed for included patients. The attending physicians determined the frequency of examinations for the patients.

### Statistical analysis 

Statistical analysis was performed using SPSS 22.0. Demographic data (gender, comorbidities, etc.) were analyzed descriptively. For continuous variables, we checked them with the normal distribution test. The variables were presented as Mean ± S.D. or Median (IQR). The binary variable was shown as frequency and composition ratio description. Mann Whitney U test, χ² test, or Fisher's exact test were used to compare differences between coagulopathy patients and non-coagulopathy patients where appropriate. To explore the risk factors associated with acute liver injury and renal dysfunction, univariable and multivariable logistic regression models were used. We included 198 patients with complete data for liver, renal and coagulation function tests (29 overt DIC, 36 non-overt DIC and 133 non-coagulopathy patients) in the logistic regression model. Patients had IL-6 tests (142 of 198 patients) and PCT tests (165 of 198 patients) were recruited in the logistic regression model. A two-sided α of less than 0. 05 was considered statistically significant. Statistical analyses were done using the SPSS software (version 22.0).

## Results

### Clinical and laboratory characteristics of COVID-19 patients with or without coagulopathy

Of the 198 COVID-19 patients enrolled in the current study, 65 patients (32.8 %) developed coagulopathy, of which 29 (14.6 %) were diagnosed with overt DIC (score ≥ 5) and 36 (18.2 %) were diagnosed with non-overt DIC (3 ≤ score ≤ 4) according to the ISTH scoring system. As shown in Table 1(Tab. 1), the median ages of overt and non-overt DIC patients were 70 (55.0-88.0) and 59.5 (33.0-89.0) years old, respectively, which were both significantly older than the patients without coagulopathy (43.0 (24.0-88.0) years). Males were more predominant in both overt and non-overt DIC patients (25; 86 % and 27; 75 %, respectively, versus 62; 47 % in non-coagulopathy patients). Nearly half of the patients (43 %) had at least one comorbidity. Hypertension and diabetes were the most common comorbidities, which were more frequent among non-overt patients (39 %, 19 %) and overt DIC patients (34 %, 24 %) than the patients without coagulopathy (14 %, 9 %). 

The median times from admission to hospital to the development into coagulopathy onset were 5 (0, 16) days for non-overt DIC and 7 (0, 30) days for overt DIC, respectively. Coagulation was monitored during whole period of hospitalization. Table 1(Tab. 1) shows the coagulation parameters of the overt DIC and non-overt DIC patients on the day of coagulopathy onset, the last hematological examination data right before the onset of coagulopathy, the highest values of CRP, PCT and IL-6 before coagulopathy occurrence, and the measurements of arterial oxygen partial pressure (PO_2_) and liver/kidney functional enzymes after coagulopathy onset. For non-coagulopathy patients, we collected the hematological and coagulation examination parameters closest to the 7^th^ day, the highest CRP, PCT and IL-6 values during hospitalization, and the measures of PaO_2_ and liver/kidney functional enzymes that deviate the most from the standard values during hospitalization.

The laboratory findings for three groups were also summarized in Table 1(Tab. 1). The median platelet counts were significantly lower in overt-DIC patients (45 (6,152)) than the patients with non-overt DIC (149 (64,425) and patients without coagulopathy (196 (83,438)). Sixteen (55 %) overt DIC patients had platelet number below 50×10^9^/L, while none in non-overt DIC group or non-coagulopathy group had platelet number below 50×10^9^/L. Fibrinogen (FIB) levels were significantly declined in overt DIC patients (2.08 (0.25,4.73)), while remained high and were not different in other two groups. More than half (57 %) of the patients without coagulopathy had D-dimer levels exceeding the normal range (0-0.5 μg/mL) during hospitalization, among which 32 (24 %) patients had a level of D-dimer above 1 μg/mL; while 27 (93 %) of overt DIC patients and 34 (94 %) non-overt DIC patients had D-dimer levels above 1 μg/mL. Monocyte counts in overt DIC patients (0.15 (0.0,0.94)) were significantly lower than the patients with non-overt DIC (0.27 (0.0,17.5)) and patients without coagulopathy (0.36 (0.1,7.0)). Sixteen (55 %) overt DIC patients and 22 (61 %) non-overt DIC patients developed leukocytosis (white blood cell count ≥ 10×10^9^ /L), and 10 (34 %) overt DIC patients and 9 (25 %) non-overt DIC patients developed neutrophilia, which were much higher proportions than those in non-coagulopathy patients (13 (9 %) and 3 (2 %)). In overt DIC patients and non-overt DIC patients, levels of inflammation-related indicators, such as PCT, high sensitivity CRP and IL-6 were significantly higher than those in non-coagulopathy patients. 

### Coagulopathy was associated with multi-organ dysfunction in COVID-19

Forty-five percent (13 out of 29) of the patients with overt DIC developed acute liver injury and 55 % developed renal dysfunction, which were much higher than those in non-overt DIC and non-coagulopathy patients (Table 1(Tab. 1)). Compared with the patients without coagulopathy, the patients with coagulopathy, including overt DIC and non-overt DIC patients, had significantly higher concentrations of liver and renal functional enzymes (Table 1(Tab. 1)). The patients with coagulopathy had persistent and lower albumin concentrations than the patients without coagulopathy. Twenty six (90 %) patients with overt DIC and 36 (89 %) patients with non-overt DIC developed hypoalbuminemia during hospitalization, while only 35 (26 %) patients without coagulopathy had hypoalbuminemia. Parameters of liver damage, such as alanine aminotransferase (ALT), aspartate aminotransferase (AST), γ-glutamyl transpeptidase (GGT), total bilirubin (TBIL) and direct bilirubin (DBIL) were markedly increased in both overt DIC and non-overt DIC patients, compared to the patients without coagulopathy. More than half of the overt DIC patients and non-overt DIC patients had higher ALT and AST concentrations. The concentrations of the other liver functional enzymes, such as alkaline phosphatase (ALP), γ-glutamyl transpeptidase (GGT) and total bile acid (TBA) were significantly higher in overt DIC patients than the other two groups. The concentrations of kidney functional index, urea nitrogen (BUN) and creatinine (Cre), were markedly higher in patients with overt DIC and non-overt DIC than patients without coagulopathy. 

We further analyzed the association between clinical characteristics and the development of multi-organ dysfunction. In univariable analysis, increased CRP concentrations and incidence of coagulopathy were associated with acute liver injury (Table 2(Tab. 2)). Odds of abnormal renal function were higher in patients with diabetes and hypertension. Increased age, male gender, increased CRP or PCT levels, and coagulopathy were also associated with abnormal renal function (Table 2(Tab. 2)). To note that, even if there was a statistical difference in IL-6 concentrations between patients with coagulopathy and patients without coagulopathy, IL-6 was not a risk factor for liver and kidney dysfunction in our logistic regression model.

In multivariable logistic regression model, the occurrence of overt DIC and elevated CRP levels were associated with increased odds of acute liver injury (Table 2(Tab. 2)). Demographic characteristics, including the older age, male gender and the history of diabetes, were associated with increased risk of renal dysfunction. The incidence of coagulopathy and elevated CRP concentrations were also associated with the increased odds of renal dysfunction (Table 2(Tab. 2)). These data suggest that coagulopathy is associated with multi-organ dysfunction in COVID-19. 

**Figure 1:**
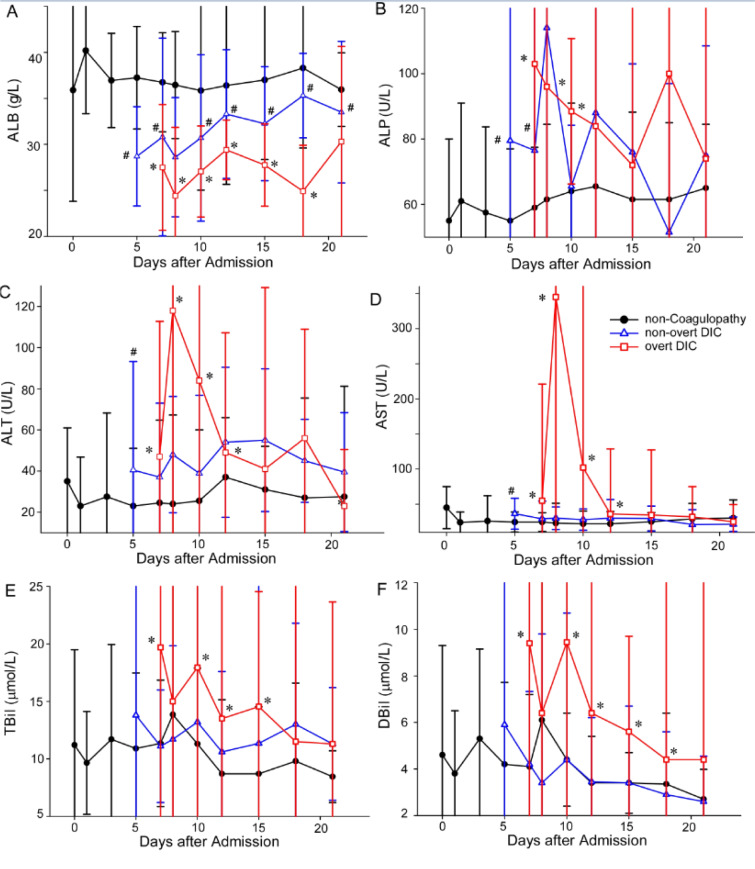
Temporal changes of laboratory parameters related to liver functions in COVID-19 patients with or without coagulopathy after DIC occurrence. Figure 1 shows dynamic changes of laboratory parameters related to liver functions, including albumin (A), alkaline phosphatase (B), alanine aminotransferase (C), aspartate aminotransferase (D), total bilirubin (E), and direct bilirubinand (F) in 198 patients (133 non-coagulopathy patients, 36 non-overt patients and 29 overt-DIC patients) from the day of coagulopathy onset (for coagulopathy patients) or the day of admission (for non-coagulopathy) to discharged or died. *P < 0.05 for overt DIC patients vs non-coagulopathy patients;^ #^*P *< 0.05 for non-overt DIC patients vs non-coagulopathy patients. ALB=albumin, ALP= alkaline phosphatase, ALT= alanine aminotransferase, AST= aspartate aminotransferase, TBil=total bilirubin, DBil= direct bilirubinand.

**Figure 2:**
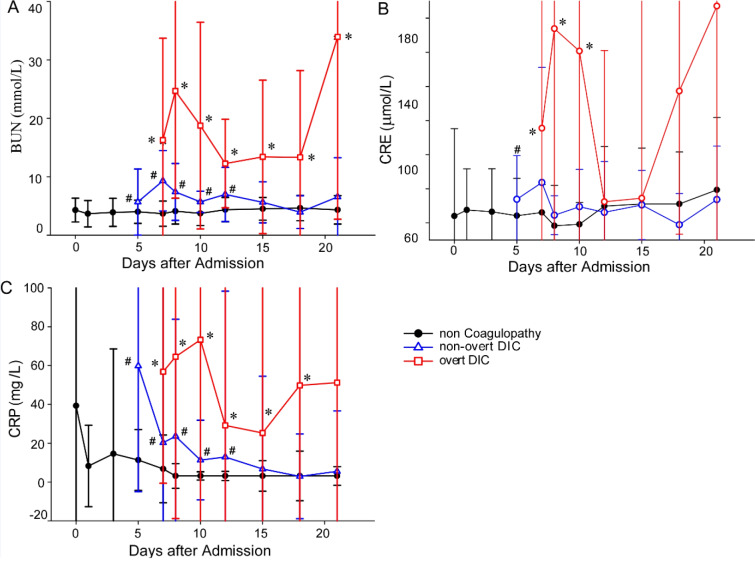
Temporal changes of renal and inflammation indices in COVID-19 patients with or without coagulopathy after DIC occurrence. Figure 2 shows dynamic changes of biomarkers of kidney functions and inflammation, including blood urea nitrogen (A), creatinine (B), and high sensitivity C-reactive protein (C) in 198 patients (133 non-coagulopathy patients, 36 non-overt patients and 29 overt-DIC patients) from the day of coagulopathy onset (for coagulopathy patients) or the day of admission (for non-coagulopathy) to discharged or died. *P < 0.05 for overt DIC patients vs non-coagulopathy patients;^ #^*P *< 0.05 for non-overt DIC patients vs non-coagulopathy patients. BUN=blood urea nitrogen, CREA=creatinine, CRP=high sensitivity C-reactive protein.

To explore the association of coagulation and multiple organ failure in COVID, the major laboratory markers of renal/liver functions, systemic inflammation, and coagulopathy occurrence were tracked. As shown in Figure 1(Fig. 1) and 2(Fig. 2), the median time from the admission to hospital to the onset of non-overt DIC and overt DIC was 5 (0-16) days and 7 (0-30) days, respectively. In patients with coagulopathy, the concentrations of ALP, ALT and AST increased sharply when coagulation occurred. The TBIL and DBIL concentrations also significantly increased when coagulation occurred. The concentrations of albumin decreased significantly accompanying the deterioration of patients' coagulation function. CRP concentrations of the patients with coagulopathy were higher than patients without coagulopathy, but showing a downward trend after the occurrence of DIC. The blood urea nitrogen and creatinine concentrations in patients with coagulopathy were also higher than the patients without coagulopathy.

### Coagulopathy was associated with respiratory function dysfunction in COVID-19 

Of the entire cohort, 56 patients received blood gas examination, including 11 patients with overt DIC, 14 with non-overt DIC, and 31 without coagulopathy. Eight of 11 (73 %) patients with overt DIC and 10 of 14 (71 %) with non-overt DIC had developed into acute respiratory distress syndrome (ARDS) during hospitalization, while only 2 of 35 (6 %) patients without coagulopathy had ARDS. The PaO_2_ was markedly lower in both overt DIC and non-overt DIC groups than the patients without coagulopathy (Table 2(Tab. 2)), suggesting that lung function is damaged in the patients with coagulopathy. 

In the current study, 56 of 198 patients received blood gas examination, and 20 of them were diagnosed with ARDS. In the univariable logistic regression analysis, elevated white blood cell counts, the occurrence of coagulopathy and overt DIC were associated with ARDS, while the multivariable logistic regression model showed that only coagulopathy was associated with the increased odds of ARDS (Table 2(Tab. 2)). 

Multi-organ dysfunction occurred after the onset of DIC in COVID-19.

To determine whether coagulopathy contributes to organ damage, we compared organ function of the patients prior to the onset of coagulopathy to the patients without coagulopathy. In total 65 patients with coagulopathy, only 9 of 29 patients with overt DIC and 13 of 36 with non-overt DIC developed coagulopathy after admission to hospital. We collected laboratory data that were measured within 2 days post admission to calculate the baseline level of each parameter (Table 3(Tab. 3)). 8 of 133 patients without coagulopathy did not have their laboratory examinations until day 3 of admission. Therefore, we compared the organ function of the 9 patients with overt DIC and 13 of 36 with non-overt DIC to 125 patients without coagulopathy. Prior to onset of coagulopathy, the median platelets counts of overt DIC group were 125 (61, 230), which were significantly lower than non-coagulopathy patients, and there were no differences in platelet count between non-overt DIC patients and non-coagulopathy patients on admission. The baseline lymphocyte counts of the non-overt DIC patients (0.67 (0.4, 2.31)) and overt DIC patients (0.67 (0.01, 1.22)) were significantly lower than non-coagulopathy patients (1.265 (0.25, 4.49)) on admission. The neutrophil counts of overt DIC patients (4.44 (0.02, 26.3)) and non-overt DIC patients (5.86 (0, 11.86)) were statistically higher than that of non-coagulopathy patients (2.195 (0.00, 19.98)) on admission. The counts of white blood cells (6.75 (2.35, 12.7)) and levels of D-dimer (0.92 (0.2, 2.29)) in non-overt DIC patients were markedly higher than non-coagulopathy patients, but there were no statistical differences in white blood cells and D-dimer concentrations between overt DIC patients and non-coagulopathy patients on admission. Concentrations of CRP in overt DIC patients were markedly elevated compared to non-coagulopathy patients. Only 4 of 9 overt DIC patients, 8 of 13 non-overt DIC patients, and 62 of 123 non-coagulopathy patients received IL-6 tests. The concentration of IL-6 was markedly increased in coagulopathy group, including overt DIC and non-overt DIC patients, compared with non-coagulopathy group on admission. There was no statistical difference in PCT concentrations between coagulopathy and non-coagulopathy groups. Significant decreased albumin level and higher blood urea nitrogen level were observed in non-overt DIC baseline; while the level of γ-glutamyl transpeptidase in overt DIC patients was significantly increased as compared to non-coagulopathy patients. The concentrations of the markers for liver and renal damage, including ALT, AST, ALP, total bile acid and creatinine were similar between coagulopathy patients and non-coagulopathy patients on admission (Table 3(Tab. 3); Supplementary Figure 1 and 2). These data indicate that multiple organ damage occurs after the onset of coagulopathy, suggesting the coagulopathy may be at least one of causes of multiple organ damage in COVID-19.

### Potential mechanisms of coagulopathy in COVID-19

Next, we tried to identify the risk factors associated with coagulopathy in COVID-19 patients. The multivariate analysis indicates the elevated CRP concentration was the risk factor of incidence of overt DIC (Supplementary Table 1); while hypertension, leukocytosis and elevated CRP concentrations were associated with coagulopathy (Table 4(Tab. 4)). Although monocyte counts were not significantly associated with overt-DIC or coagulopathy, profoundly declined monocyte counts were observed in overt-DIC group from 0.37 (0.12, 1.75) on admission day to 0.15 (0.0, 0.94) on the day of overt DIC onset (Supplementary Table 2). As shown in Table 1(Tab. 1) and 3(Tab. 3), increases of IL-6 were detected in both non-overt and overt DIC groups on admission and around the time of coagulopathy occurrence compared with non-coagulopathy patients; in addition, no differences in IL-6 concentrations were observed before and after coagulopathy occurrence in Supplementary Table 2. IL-6 was not associated with COVID-19-induced overt DIC or coagulopathy by the univariable logistic regression analysis (Table 4(Tab. 4) and Supplementary Table 1). There were no differences in PCT levels and white blood cells counts before and after coagulopathy. Only 3 of 10 overt DIC patients had procalcitonin concentrations above 2 ng/mL, and none patients' procalcitonin levels exceeded 2 ng/mL in non-overt DIC patients, these data indicated most of coagulopathy were triggered by COVID-19 without bacterial infection. 

### Patients with coagulopathy had reduced survival rate

**Figure 3:**
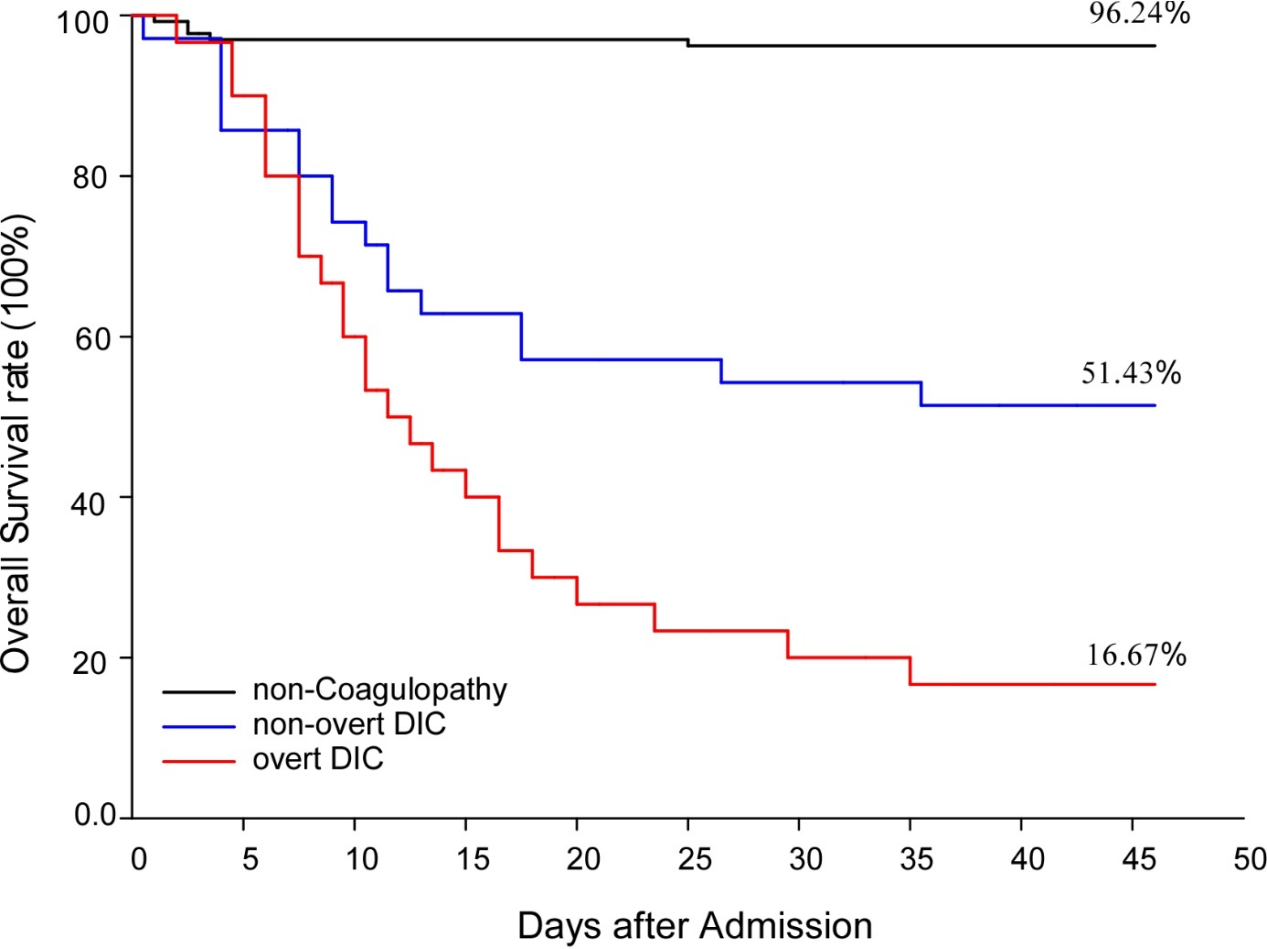
Survival curves of COVID-19 patients with or without coagulopathy. Figure 3 illustrated survival rates of overt DIC patients, non-overt patients or non-coagulopathy patients during hospitalization.

Survival curves for the overt-DIC, non-overt DIC and non-coagulopathy groups are shown in Figure 3(Fig. 3). Twenty five of 30 (83.33 %) patients with overt-DIC and 17 of 35 (48.57 %) with non-overt DIC died during hospitalization, while only 5 of 133 (3.75 %) without coagulopathy died. Multivariable logistic regression analysis suggested that coagulopathy (17 (6.196, 46.642), *p *< 0.01) and DIC (9.529 (2.435, 37.300), *p < *0.01) were significantly associated with increased mortality. The median time from the occurrence of overt DIC to death was 3 (1,15) days, and the median time from the onset of non-overt DIC to death was 6 (0,29) days. 

## Discussion

In this retrospective cohort study, we showed that occurrence of coagulopathy is associated with multi-organ dysfunction and ARDS, as well as an increasing mortality rate in COVID-19. History of hypertension, leukocytosis and elevated CRP concentrations were associated with higher odds of coagulopathy. Additionally, elevated levels of blood IL-6 and CRP occurred more frequently in patients with coagulopathy, and monocyte counts were significantly decreased when coagulopathy occurs.

In our study, twenty nine (15 %) patients developed overt DIC, and 89 % non-survival COVID-19 patients had coagulopathy, including 36 % non-overt DIC patients and 53 % overt DIC patients. Similar as previous studies, mortality rate is significantly in COVID-19 patients who had coagulopathy, suggesting that coagulopathy is a risk factor in COVID-19. The overall incidence of DIC in COVID-19 is not clear. It seems that the incidence of coagulopathy in our study is higher than some previous studies (Chen et al., 2020[3]). This discrepancy seems to be due to the selectivity of patients. In our study, we selected hospitalized patients from the Infectious Care unit and ICU wards. It has been reported that the platelet counts are similar between ICU care and non-ICU care patients on admission (Huang et al., 2020[10]), and only 20 % of non-survivors have platelet counts below 100x10^9^/ L3. Thus, the count of platelet is regarded as an insensitive marker for disease severity of COVID-19 patients (Zhou et al., 2020[28]; Guan et al., 2020[8]; Huang et al., 2020[10]; Tang et al., 2020[17]). We found that platelet counts declined dramatically after the onset of coagulopathy in both non-overt DIC patients and overt DIC patients from the admission to the occurrence of overt DIC. Our data suggest that a sharp decrease in platelet count may be a valuable indicator of the occurrence of coagulopathy in COVID-19. In addition, all patients with coagulopathy had increased D-dimer concentrations, about 55 % of overt DIC patients and only 2 % of non-overt DIC patients had PT exceeded 3 seconds; none of non-overt DIC patients and 27 % of overt DIC patients had fibrinogen concentrations decreasing to the level below 1.0 g/L. 

There are two distinct types of DIC: one is characterized by a strong coagulation activation and suppressed fibrinolysis, resulting in fibrin deposition and prothrombotic DIC, the other type is based on excessive fibrinolysis, manifested by severe bleeding complications (Levi, 2018[13]). It has been reported that most COVID-19 patients are under hypercoagulable and hyperfibrinolysis state, which are manifested by declined PT and APTT (Chen et al., 2020[2]) and elevated D-dimer levels (Guan et al., 2020[8]; Huang et al., 2020[10]; Wang et al., 2020[19]; Lantner and Reisman, 1989[11]). We found that in the non-overt DIC group, 94 % had D-dimer above 3 μg/ml and 61 % patients had increased FDP concentration; however, only 25 % patients had thrombocytopenia and 11 % patients developed prolonged PT or APTT, suggesting a hypercoagulable and hyperfibrinolysis state in the context of non-overt DIC. Thus, anticoagulant and antifibrinolytic therapy may be necessary for non-overt DIC patients. In contrast, although most of the overt DIC patients a had high level of D-dimer (over 3 μg/ml) and increased FDP, which suggested a secondary hyperfibrinolysis condition, 75 % of them exerted a significant decline of platelet counts and 31 % showed decreased fibrinogen levels, indicating that the ongoing thrombosis may eventually lead to hypocoagulable in overt DIC patients. Anticoagulant treatment such as heparin (Tang et al., 2020[17]) and antifibrinolytic therapy tPA (Wang et al., 2020[20]) have been proven to be beneficial for COVID-19 patients with ARDS and effectively reduced mortality. 

We recently reported that tissue factor released from pyroptotic macrophages and monocytes plays a critical role in triggering DIC in sepsis (Pandeya et al., 2019[14]). We found that the monocyte counts significantly decreased prior to the onset of coagulopathy (Wu et al., 2019[24]). Previously, older age and decreased lymphocyte counts have been reported as important independent predictors of mortality in COVID-19 patients (Zhou et al., 2020[28]; Wang et al., 2020[19]), and decreases of lymphocytes were also observed in COVID-19 patients with venous thromboembolism (Cui et al., 2020[5]). The current study showed that COVID-19 patients with coagulopathy had significantly decreased lymphocyte counts and the average age was older than those without coagulopathy on admission. Leucocytes, including monocytes and neutrophils, play critical roles in the innate immune host defense to pathogen invasion and immunothrombosis. Compared to the patients without coagulopathy, the increase of neutrophil counts was observed in patients with coagulopathy. Neutrophils were thought as the main source of chemokines and cytokines, and increasing neutrophil extracellular traps are thought to contribute to the severity of COVID-19 (Barnes et al., 2020[1]). Increase in neutrophils may be caused by subsequent bacterial infection. We are not sure whether bacterial infection also contributes to the development of DIC in COVID-19. 

It has been well known that multiple organ damage is a common and lethal complication in severe COVID-19 patients (Chen et al., 2020[3]; Zhou et al., 2020[28]; Guan et al., 2020[8]; Huang et al., 2020[10]; Deng et al., 2020[7]; Lantner and Reisman, 1989[11]; Wang et al., 2020[21]). Although ACE2 receptor is expressed in cholangiocytes (Rajapaksha et al., 2019[15]) and kidney (Letko et al., 2020[12]; Rella et al., 2007[16]), which can facilitate the binding and endocytosis of the virus into liver and kidney, pathological analysis of liver tissue from a COVID-19 patient showed that viral inclusions were not observed in the liver (Xu et al., 2020[25]), and no evidence of coronavirus infection was observed in renal tissue (Yao et al., 2020[26]). Our data show that patients prior to coagulopathy onset had similar levels of hepatic and renal enzymes as patients without coagulopathy. The patients exhibited significant damage of hepatic and renal function right after the onset of coagulopathy. These data suggest that coagulopathy is at least one of the major causes of multiple organ damage in COVID-19. Immune-mediated inflammation, such as cytokine storm and pneumonia-associated hypoxia, is generally considered to be the cause of organ damage. In the present study, we also found that systemic inflammatory indicators C-reactive protein and IL-6 were persistent higher in coagulopathy patients than those in non-coagulopathy patients from the admission day to the coagulopathy onset. However, our multivariable regression analysis indicates that coagulopathy, but not elevated IL-6, is associated with increasing odds of acute liver injury, renal dysfunction and ARDS. The pathological findings of COVID-19 also revealed inflammatory infiltration in lung and heart, but not in liver or kidney; additionally, hyaline thrombus in capillaries were observed in renal, liver and lung (Xu et al., 2020[25]; Yao et al., 2020[26]). Consistent with our finding, a previous study suggests that elevated coagulation function indices (PT and D-dimer) are significantly associated with higher risk of ARDS (Wu et al., 2020[23]). Except coagulopathy, other mechanisms may also contribute to multiple organ damage in COVID. In this regard, it has been reported that IL-6 is positively correlated with disease severity of COVID-19 (Zhang et al., 2020[27]; Henry et al., 2020[9]).

The current study has certain limitations. First, not all of the 198 patients received IL-6 and procalcitonin tests, therefore the roles of IL-6 and procalcitonin in coagulopathy or multi-organ dysfunction might be underestimated due to the limited sample size. Second, not all of the 198 patients received blood gas detection during hospitalization, which was determined by physicians based on the breathing status. Thus, the effect of coagulation dysfunction on lung function cannot be well determined. Last, not all the patients had laboratory examination data at all time points in the dynamic graphs, so we combined the examination values of patients on the specified date and the day after that. However, from the current data we feel confident to conclude the coagulopathy is one of the major causes leading to multiple organ damage in COVID-19. Further studies are needed to identify the mechanism by which the severe acute respiratory syndrome coronavirus 2 (SARS-CoV-2) triggers activation of coagulation.

## Notes

Rui Zhang and Yani Liu contributed equally as first authors.

Zhenyu Li and Shaojun Shi (Department of Pharmacy, Union Hospital, Tongji Medical College, Huazhong University of Science and Technology, 1277 Jiefang Avenue, Wuhan 430022, PR China; Phone: +86-13971292838, Fax: +86-027–8572–6192, E-mail: sjshicn@163.com) contributed equally as corresponding authors.

## Authorship

R.Z., Y.Z., Z.L. and S.S. conceived and designed the study. R.Z., Y.L., J.Z. and W.Y. collected the clinical data. R.Z., Y.L., B.Z. and C.W. analyzed the data. All authors wrote, corrected and read the manuscript.

The data that support the findings of this study are available on request from the corresponding authors.

## Acknowledgement

This work was supported by the National Natural Science Foundation of China (81874326), Chinese Medicine Research Project of Health Commission of Hubei Province (ZY2019Z004); National Key R&D Program of China (2017YFC0909900).

## Conflict of interest

The authors declare no conflicts of interest.

## Supplementary Material

Supplementar information

## Figures and Tables

**Table 1:**
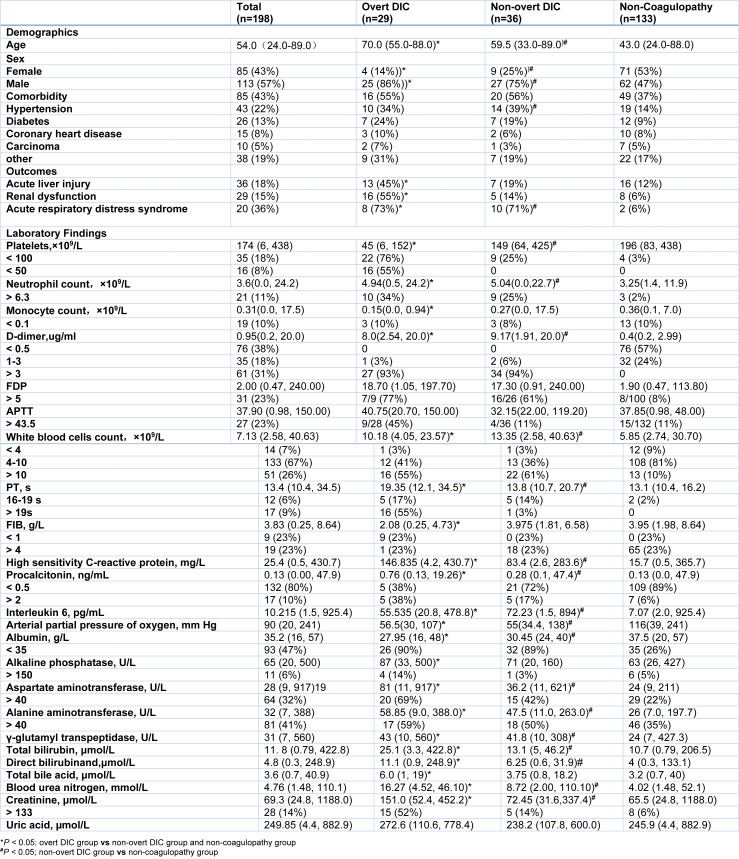
Demographics and clinical characteristics of COVID-19 patients with or without coagulopathy

**Table 2:**
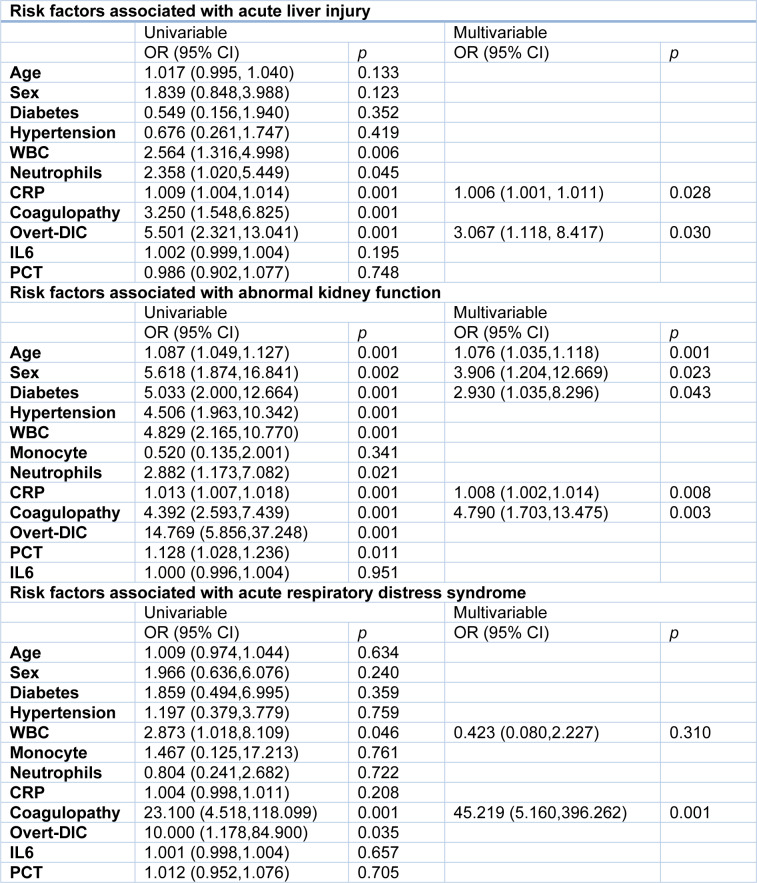
Risk factors associated with organ function

**Table 3:**
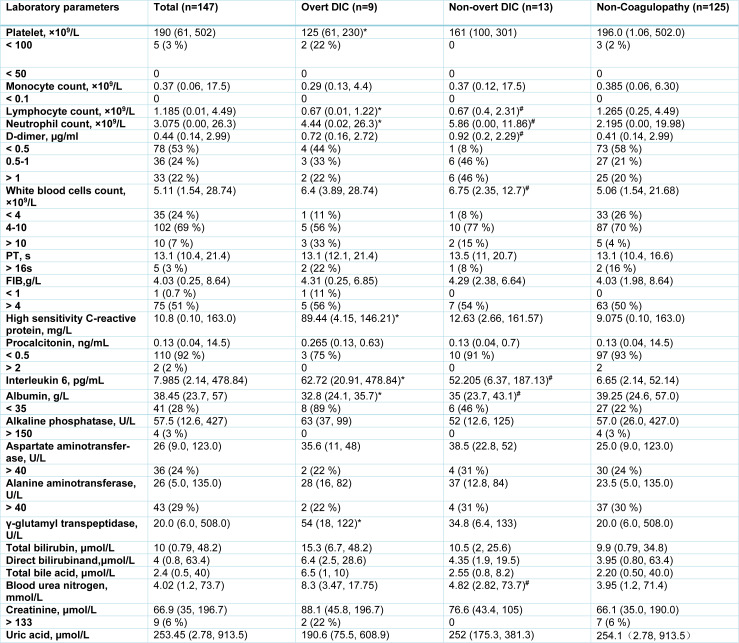
Baseline characteristics of COVID-19 patients with or without coagulopathy on admission

**Table 4:**
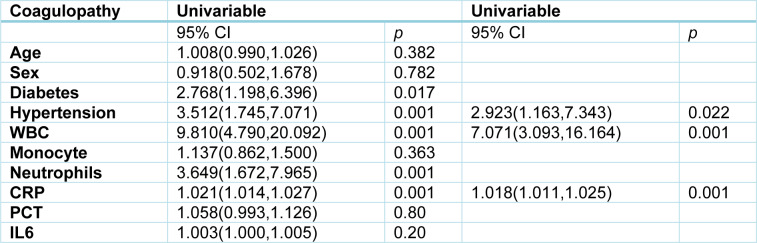
Risk factors associated with coagulopathy
