# Supplementary material for: Coagulopathy is associated with multiple organ damage and prognosis of COVID-19
Source: EXCLI J. 2021 Jan 25;20:174–91. doi: 10.17179/excli2020-2853 (PMC7868640; doi:10.17179/excli2020-2853)
Supplement: Supplementar information [file EXCLI-20-174-s-001.pdf]

EXCLI Journal 2021;20:174-191 – ISSN 1611-2156
Received: August 30, 2020, accepted: December 09, 2020, published: January 25, 2021

Supplementary information to:

Original article:

COAGULOPATHY IS ASSOCIATED WITH MULTIPLE ORGAN DAMAGE AND PROGNOSIS OF COVID-19

Rui Zhang[1*], Yani Liu[1*], Bei Zhang[2], Congqing Wu[3], Jinping Zhou[1], Yu Zhang[1], Wei Yang[4], Zhenyu Li[3**], Shaojun Shi[1**]

1 Department of Pharmacy, Union Hospital, Tongji Medical College, Huazhong University of Science and Technology, Wuhan 430022, PR China
2 College of Public Health, Shaanxi University of Chinese Medicine, Xianyang 712046, PR China
3 Saha Cardiovascular Research Center, College of Medicine, University of Kentucky, Lexington, KY40536, USA
4 Department of Nutrition and Food Hygiene, Hubei Key Laboratory of Food Nutrition and Safety, School of Public Health, Tongji Medical College, Huazhong University of Science and Technology, 13 Hangkong Road, Wuhan 430030, PR China

* These authors contributed equally to this work.

** **Corresponding authors:** Zhenyu Li, Saha Cardiovascular Research Center, College of Medicine, University of Kentucky, Lexington, KY40536, USA, E-mail: zhenyuli08@uky.edu
Shaojun Shi, Department of Pharmacy, Union Hospital, Tongji Medical College, Huazhong University of Science and Technology, 1277 Jiefang Avenue, Wuhan 430022, PR China; Phone: +86-13971292838; Fax: +86-027–8572–6192; E-mail: sjshicn@163.com

https://orcid.org/0000-0001-7245-7200 (Zhenyu Li)
https://orcid.org/0000-0002-4942-4360 (Shaojun Shi)

http://dx.doi.org/10.17179/excli2020-2853

This is an Open Access article distributed under the terms of the Creative Commons Attribution License (http://creativecommons.org/licenses/by/4.0/).

EXCLI Journal 2021;20:174-191 – ISSN 1611-2156
Received: August 30, 2020, accepted: December 09, 2020, published: January 25, 2021

Supplementary Table 1: Risk factors associated with overt DIC

Overt DIC	Univariable		Multivariable	
	95% CI	*p*	95% CI	*p*
Age	1.662 (0.711,3.887)	0.241		
Sex	1.005 (0.981,1.029)	0.701		
Diabetes	2.512 (0.947,6.662)	0.064		
Hypertension	2.169 (0.922,5.100)	0.076		
WBC	3.986 (1.838,8.643)	0.001	2.308 (0.876,6.081)	0.091
Monocyte	1.161 (0.983,1.371)	0.079		
Neutrophil	2.882 (1.173,7.082)	0.021		
CRP	1.018 (1.011,1.025)	0.001	1.017 (1.010,1.024)	0.001
PCT	1.038 (0.977,1.102)	0.229		
IL6	1.002 (0.999,1.005)	0.176		

Supplementary Table 2: Potential mechanisms of coagulopathy in COVID-19

	**Before overt DIC**	**After overt DIC**	**Before non-overt DIC**	**After non-overt DIC**
Interleukin 6, pg/mL	33.29 (18.72, 478.84)	34.29 (10.5, 76.86)	69.00 (4.04, 261.90)	21.03 (1.50, 411.70)
White blood cells count, $\times10^9$/L	13.58 (4.05, 36.74)	15.46 (0.74, 35.41)	9.94 (2.35, 24.28)	10.70 (2.38, 30.91)
> 10	14 (67 %)	19 (70 %)	4 (36 %)	20 (63 %)
Procalcitonin, ng/mL	1.34 (0.08, 11.09)	0.89 (0.13, 19.26)	0.26 (0.13, 0.83)	0.13 (0.06, 31.90)
> 2	3 (33 %)	3 (37 %)	0	4 (16 %)
	**Before overt DIC**	**Around overt DIC**	**Before non-overt DIC**	**Around non-overt DIC**
Monocyte count, $\times10^9$/L	0.37 (0.12, 1.75)	0.15 (0.00, 0.94)*	0.29 (0.01, 4.40)	0.21 (0.00, 0.40)

EXCLI Journal 2021;20:174-191 – ISSN 1611-2156
Received: August 30, 2020, accepted: December 09, 2020, published: January 25, 2021

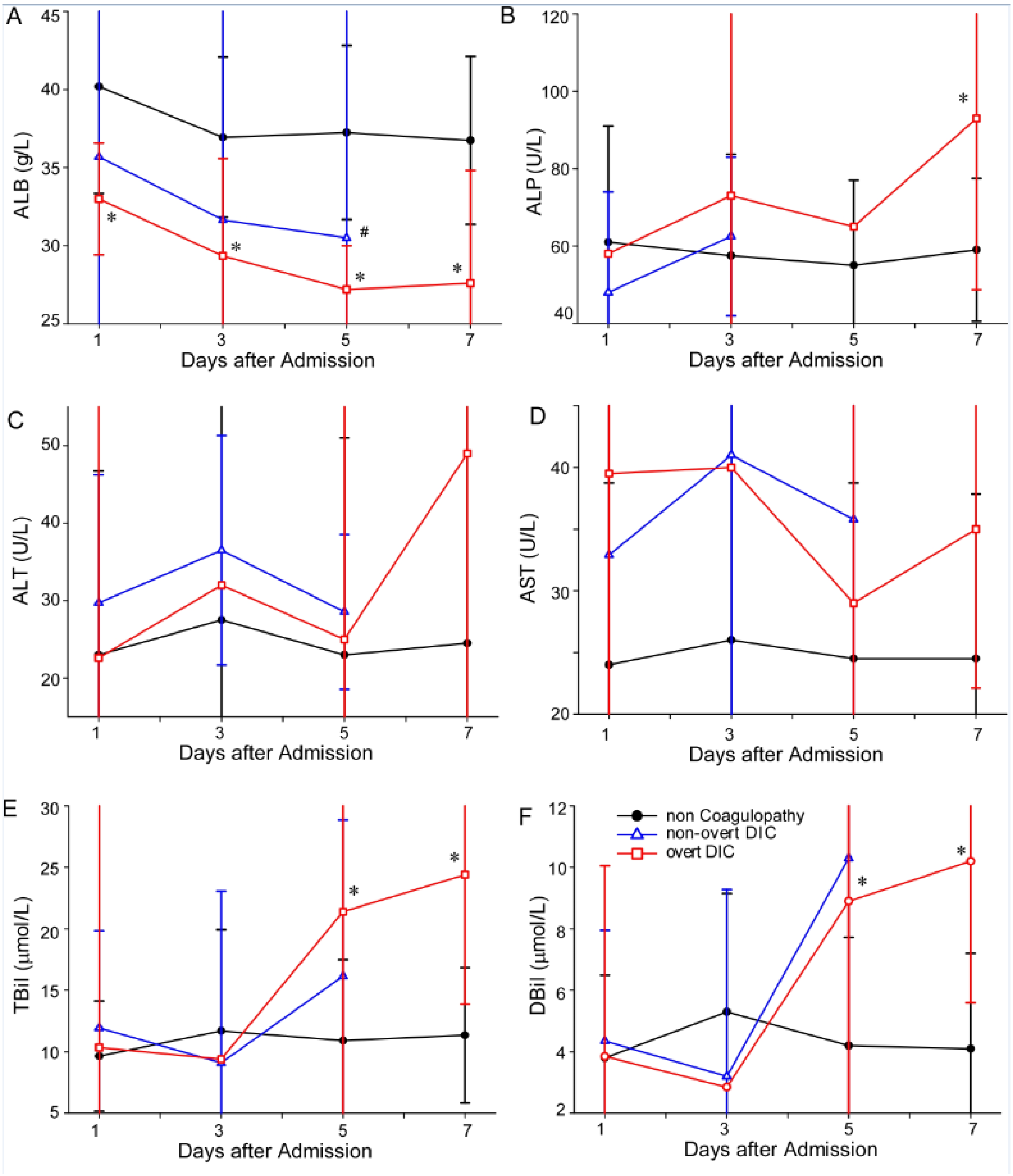

Supplementary Figure 1: Temporal changes of laboratory parameters related to liver functions in COVID-19 patients with or without coagulopathy before DIC occurrence

Supplementary Figure 1 shows dynamic changes of laboratory parameters related to liver functions, including albumin (**A**), alkaline phosphatase (**B**), alanine aminotransferase (**C**), aspartate aminotransferase (**D**), total bilirubin (**E**), and direct bilirubin (**F**) in 147 patients (125 non-coagulopathy patients, 13 non-overt patients and 9 overt-DIC patients) from the day of admission to the day before coagulopathy onset (for coagulopathy patients) or the day of admission (for non-coagulopathy) to day 7. *$P < 0.05$ for overt DIC patients vs non-coagulopathy patients; #$P < 0.05$ for non-overt DIC patients vs non-coagulopathy patients. ALB=albumin, ALP=alkaline phosphatase, ALT= alanine aminotransferase, AST=aspartate aminotransferase, TBil=total bilirubin, DBil= direct bilirubinand.

EXCLI Journal 2021;20:174-191 – ISSN 1611-2156
Received: August 30, 2020, accepted: December 09, 2020, published: January 25, 2021

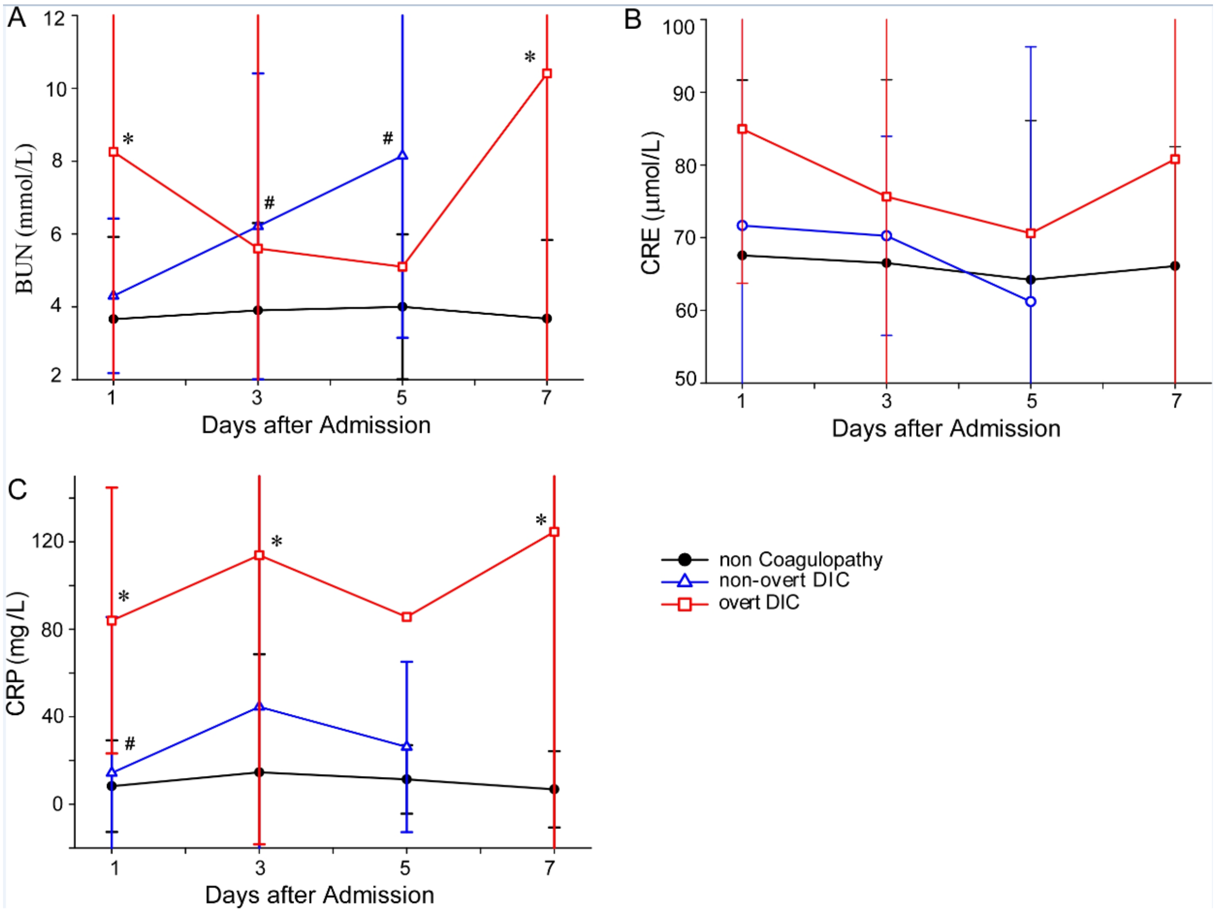

Supplementary Figure 2: Temporal changes of renal and inflammation indices in COVID-19 patients with or without coagulopathy before DIC occurrence

Supplementary Figure 2 shows dynamic changes of biomarkers of kidney functions and inflammation, including blood urea nitrogen (**A**), creatinine (**B**), and high sensitivity C-reactive protein (**C**) in 147 patients (125 non-coagulopathy patients, 13 non-overt patients and 9 overt-DIC patients) from the day of admission to the day before coagulopathy onset (for coagulopathy patients) or the day of admission (for non-coagulopathy) to day 7. *$P < 0.05$ for overt DIC patients vs non-coagulopathy patients; $^{\#}P < 0.05$ for non-overt DIC patients vs non-coagulopathy patients. BUN=blood urea nitrogen, CREA=creatinine, CRP=high sensitivity C-reactive protein.
